# Association of maternal diabetes/glycosuria and pre-pregnancy body mass index with offspring indicators of non-alcoholic fatty liver disease

**DOI:** 10.1186/s12887-016-0585-y

**Published:** 2016-03-31

**Authors:** Sumaiya Patel, Debbie A. Lawlor, Mark Callaway, Corrie Macdonald-Wallis, Naveed Sattar, Abigail Fraser

**Affiliations:** School of Social and Community Medicine, University of Bristol, Oakfield House, Oakfield Road, Bristol, UK; School of Social and Community Medicine, University of Bristol, UK & MRC Integrative Epidemiology Unit at the University of Bristol, Bristol, UK; University Hospitals Bristol NHS Foundation Trust, Bristol, UK; Institute of Cardiovascular & Medical Sciences, BHF Glasgow Cardiovascular Research Centre, Faculty of Medicine, University of Glasgow, Glasgow, UK

**Keywords:** Pregnancy diabetes, Glycosuria, Obesity, NAFLD, Fetal overnutrition, ALSPAC

## Abstract

**Background:**

Little is known about early life determinants of non-alcoholic fatty liver disease (NAFLD). We examined associations of maternal pregnancy diabetes/glycosuria and pre-pregnancy body mass index (BMI) with offspring markers of NAFLD and liver pathology and examined mediation by birthweight and concurrent offspring adiposity.

**Methods:**

We used data from a UK prospective pregnancy cohort. Offspring underwent abdominal ultrasonography (USS) at mean age 17.8 years. Outcomes included USS-assessed fatty liver, estimated liver volume and shear velocity, a variant of elastography (a marker of liver fibrosis) (*N* = 1 215) and blood-based markers of liver pathology [alanine amino transferase, aspartate amino transferase, gamma- glutamyltransferase and haptoglobin] (*N* = 2 359).

**Results:**

2.1 % (*N* = 25) of participants had USS-assessed fatty liver [maternal diabetes/glycosuria (*N* = 7) and no diabetes/glycosuria (*N* = 18)]. Maternal diabetes/glycosuria was associated with greater odds of offspring USS fatty liver in confounder adjusted models [adjusted odds ratio (aOR) 6.74 (95 % confidence interval (CI) 2.47, 18.40)] and higher shear velocity [adjusted ratio of geometric mean (aRGM):1.10 (95 % CI 1.05, 1.15)]. These associations were not mediated by offspring birthweight or concurrent adiposity. Maternal diabetes/glycosuria was not associated with liver volume or blood-based outcomes. Greater maternal pre-pregnancy BMI was associated with greater odds of offspring USS fatty liver [aOR 2.72 (95 % CI: 1.20, 6.15)], higher liver volume [aRGM 1.03 (95 % CI 1.00, 1.07)] and shear velocity [aRGM1.03 (95 % CI: 1.01, 1.06)] in confounder adjusted models. These associations were largely mediated by offspring adiposity. Maternal pre-pregnancy BMI was not consistently associated with blood-based outcomes.

**Conclusions:**

Results suggest that maternal pregnancy diabetes/glycosuria is associated with offspring NAFLD through mechanisms other than offspring’s own adiposity.

**Electronic supplementary material:**

The online version of this article (doi:10.1186/s12887-016-0585-y) contains supplementary material, which is available to authorized users.

## Background

The developmental overnutrition hypothesis suggests that maternal pregnancy diabetes and adiposity are characterized by increased delivery of fuels such as glucose, free fatty acids and amino acids to the developing fetus [1, 2]. This results in greater offspring birthweight and altered neuroendocrine, pancreatic, hepatic or musculoskeletal systems, which in turn lead to greater offspring adiposity and a more adverse cardiometabolic health profile later in life [1, 2]. Several studies have reported associations of maternal pre-pregnancy body mass index (BMI) and pregnancy diabetes with long term adverse cardiometabolic outcomes in the offspring, including obesity [3, 4], glucose metabolism and dyslipidemia [5, 6]. This includes reports based on data from the Avon Longitudinal Study of Parents and Children (ALSPAC), which we use here [7–9].

Non-alcoholic fatty liver disease (NAFLD) is closely associated with greater adiposity, hyperlipidemia and hyperinsulinemia [10], diabetes [11] and is considered the hepatic manifestation of the cluster of metabolic abnormalities linked to insulin resistance. Hence it is plausible that exposure to diabetes and increased maternal adiposity in utero may be associated with a greater risk of NAFLD later in life. In animal models, maternal high fat diets [12] and maternal obesity [13, 14] during pregnancy, predisposes the developing offspring to non-alcoholic steatohepatitis and insulin resistance.

Two recent studies in humans have examined the associations of maternal pre-pregnancy BMI [15] and gestational diabetes [16] with infant offspring intrahepatocellular lipid content (IHCL) assessed by magnetic resonance spectroscopy. Modi and colleagues [15] reported an increase of 8.6 % (95 % confidence intervals [CI], 1.1, 16.8) in IHCL at a mean age of 11.7 days per maternal BMI unit increase (*N* = 105). Although due to the small number of mothers with gestational diabetes in the study sample they were unable to examine its independent effect. Brumbaugh et al. [16] reported greater IHCL content in infants aged 1–3 weeks born to obese diabetic mothers (*N* = 10) compared to infants of non-diabetic, non-obese women (*N* = 10). However, this study did not look at the association of maternal obesity and gestational diabetes separately and whether associations seen in infants persist in older ages remains unclear.

**Fig. 1:**
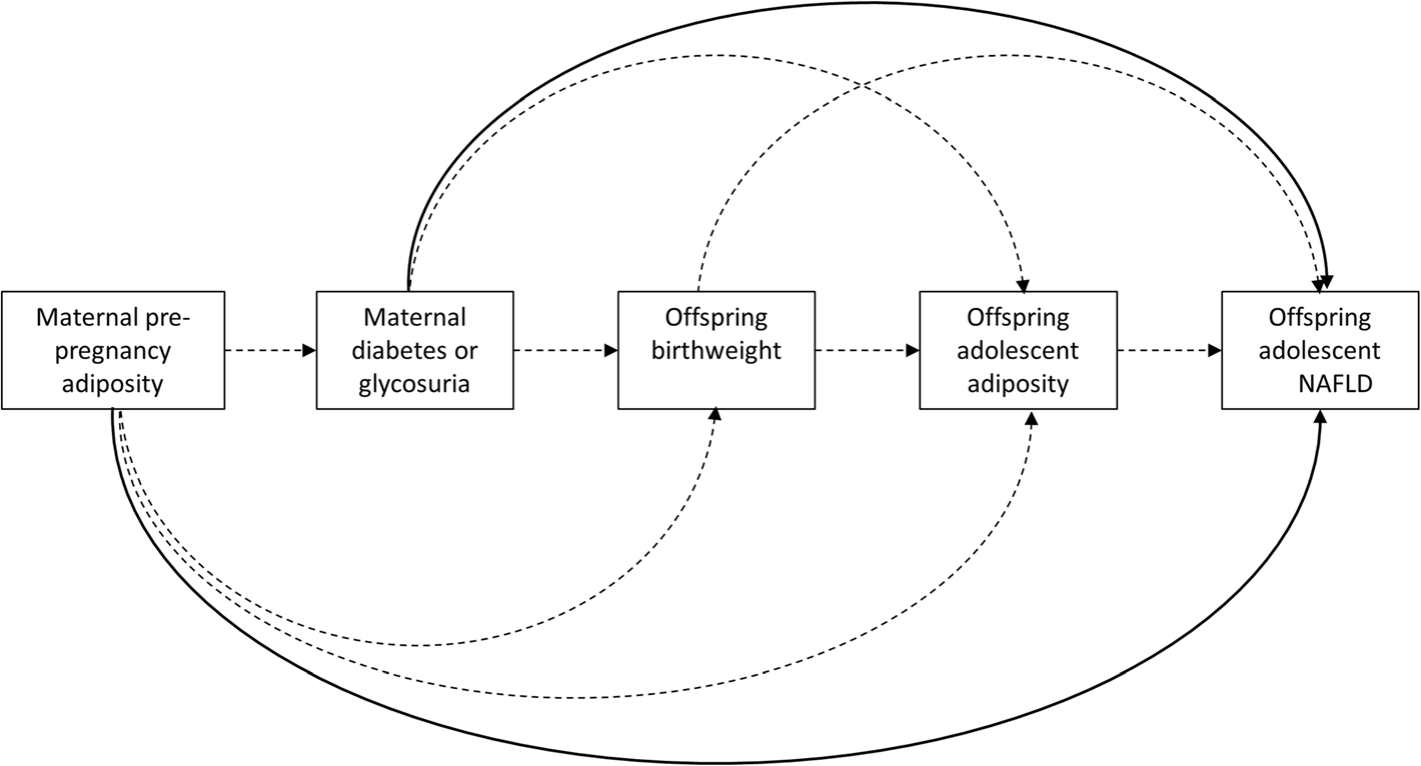
Pathways related to the associations between maternal pre-pregnancy adiposity and maternal pregnancy diabetes/glycosuria with offspring NAFLD. The dark arrows represent the main association of interest in the paper. The dashed arrows are the possible pathways for the underlying mechanism and which are examined in this study

The principal aim of this study is to examine associations of maternal pregnancy diabetes/glycosuria and pre-pregnancy BMI with offspring markers of NAFLD and liver pathology [ultrasound scan (USS) assessed fatty liver, estimated liver volume and stiffness (a marker of liver fibrosis) and fasting blood alanine amino transferase (ALT), aspartate amino transferase (AST), gamma- glutamyltransferase (GGT) and haptoglobin] in adolescence. The main outcome is USS assessed liver fat. We further aim to explore whether any associations are mediated by birthweight and/or offspring’s own concurrent adiposity. Figure 1 shows the pathways examined in this study.

## Methods

### Study participants

The Avon Longitudinal Study of Parents and Children (ALSPAC) is a prospective, population-based birth cohort study that recruited 14 541 pregnant women residing in Avon, UK, with expected dates of delivery 1st April 1991 to 31st December 1992 (http://www.alspac.bris.ac.uk) [17, 18].

**Fig. 2:**
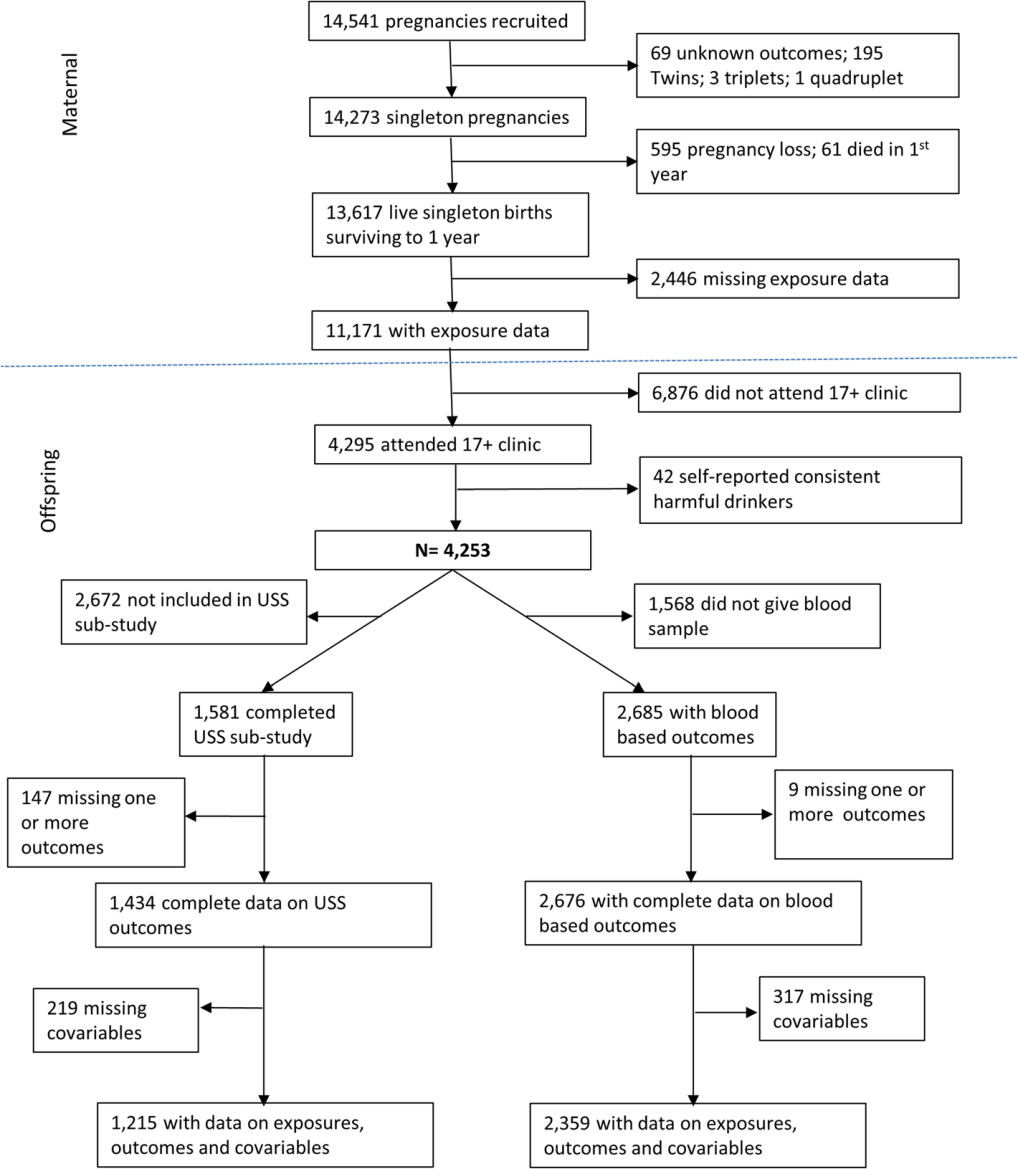
Participants’ flow diagram through the study and the numbers included in each of the main analyses

From age seven, offspring have been invited to designated research clinics. At the 17–18 year clinic a sub-sample of 1 874 offspring completed liver ultrasound scans (USS) and 3 069 had blood samples taken. For this study mother-offspring duos with data on exposures (pregnancy diabetes/glycosuria and maternal pre-pregnancy BMI) and outcomes (measures from liver USS, ALT, AST, GGT and haptoglobin) were eligible (see Fig. 2). No participants had a known history of jaundice or hepatitis, were taking medications or receiving treatment that would indicate they had hepatic disease, or were taking medication known to influence liver function. In order to remove any effect of fat infiltration in the liver due to excess alcohol intake, consistent harmful alcohol drinkers were removed from the analysis. Information on offspring’s alcohol consumption was obtained the Alcohol Use Disorders Identification Tests questionnaire [19]. This was administered to offspring at 16 years, and 17 years (at the same time as the USS assessment) and participants were scored between 0 and 20 with a score over 16 being classified as harmful alcohol consumption [19]. Consistent harmful alcohol drinkers were defined by a score of 16 or greater at both 16 and 17 years. After removal of 42 participants classified as consistent harmful drinkers 1 215 had complete data on exposures, covariables and USS outcomes and 2 359 had complete data on exposures, covariables and blood-based outcomes (ALT, AST, GGT and haptoglobin) (see Fig. 2).

Ethical approval for this study was obtained from the ALSPAC Law and Ethics Committee and the North Somerset & South Bristol National Health Service Research Ethics Committee (09/H0106/53). All participants (mothers and offspring) provided written informed consent.

### Assessment of maternal characteristics

The methods used to determine maternal pregnancy diabetes and glycosuria have been described in detail previously [7, 8]. Briefly, information on existing diabetes was collected by questionnaire from women at the time of recruitment. A standard protocol was used by research midwives to obtain information on a clinical diagnoses of gestational diabetes and glycosuria (recorded as none, trace, +, ++,+++ or more) for the index pregnancy from the woman’s antenatal and postnatal medical records. The midwives searched all aspects of these records and gestational diabetes was defined as any record of a diagnosis of gestational diabetes at any time during the pregnancy in women without existing diabetes at the start of pregnancy.

The practice in the UK at the time when mothers were recruited (April 1991 to December 1992) was for all women to be offered urine tests for glycosuria at each antenatal clinic visit. Universal screening was not undertaken and diagnostic tests for gestational diabetes, which could have included a fasting glucose or an oral glucose tolerance test, will only have been undertaken in women with established risk factors (obesity, family history of diabetes and previous history of gestational diabetes or macrosomic birth, south Asian ethnicity) or glycosuria. Glycosuria in our study was defined as a record of at least ++ (equal to 13.9 mmol/l or 250 mg/100 ml) on at least two occasions at any time during the pregnancy in the absence of existing diabetes and gestational diabetes. These three categories of maternal existing diabetes, maternal gestational diabetes and maternal glycosuria were combined in the main analysis due to the small number of offspring with outcome data in each of the categories (*N* = 7, *N* = 8 and *N* = 47 respectively). Median and interquartile range for age at diagnosis of the existing diabetes group was 12.5 years (9.0, 25.0). This early age of diagnosis suggests that this group would be predominantly type I diabetes. In this study women were categorized as having any of existing diabetes, gestational diabetes or glycosuria in pregnancy or not; this status is referred to as diabetes/glycosuria throughout the remainder of the paper.

Maternal self-reported weight and height determined by postal questionnaire at 12 week gestation were used to calculate pre-pregnancy BMI. Maternal self-reported weight correlated highly with the mother’s weight measurement at the first antenatal visit (*r* = 0.95). Maternal pre-pregnancy BMI was categorised as underweight/normal (BMI <25.0 kg/m^2^) and overweight/obese (BMI ≥25.0 kg/m^2^).

### Liver ultrasound scans and blood-based outcomes

For both the liver USS and all blood-based analyses participants were fasted overnight for those attending clinic in the morning, or for a minimum of 6 h, for those attending clinic after lunch.

### Liver ultrasound scans

A detailed description of the liver ultrasound scans has been published previously [20].

Briefly, upper abdominal USS was completed by one of four trained sonographers using a Siemens Acuson S2000 USS system, with the participant at rest in the dorsal decubitus position. Echogenicity (a marker of liver fat) was assessed during deep inspiration and recorded as present, absent or uncertain according to established protocols using the right kidney as the reference organ [21].

Acoustic radiation force impulse-imaging (ARFI) of the right lobe of the liver was used to measure liver stiffness (or fibrosis), using standard protocols [22, 23] and this was used as our main indicator of liver fibrosis. The right lobe of the liver was viewed through the intercostal space such that the pulse wave was traversing an area of at least 6 cm and was not traversing any major vascular structures and the right lobe was clearly viewed.

Estimated liver volume was assessed by three transversely (lateral to medial), from diaphragm down to the inferior pole of the liver (cranio-caudal) and back to front (posterior to anterior) sweeps through the liver.. These produced distances, which were used to calculate liver volume with the Siemens Acuson S2000 system software.

### Blood-based outcomes

Fasting blood samples were immediately spun and frozen at −80 °C. Measurements were assayed shortly (3–9 months) after with no previous freeze-thaw cycles. All assays were completed in the same laboratory (University of Glasgow). ALT, GGT and AST were measured by automated analyser with enzymatic methods and haptoglobin was measured by immunoturbidimetry.

### Other variables

Maternal age, parity, gestational age, birthweight and offspring gender were extracted from medical records. Parental occupation was used to derive household occupational social class, with each household assigned the highest parental occupational (classes I (professional/managerial) to V (unskilled manual workers), using the 1991 British Office of Population and Census Statistics classification). Maternal pre-pregnancy alcohol consumption was determined by a postal self-completed questionnaire at 18 weeks gestation and was categorised as less than or more than one glass (described in one unit portions within the questionnaire) of alcohol per week. Offspring age at clinic was recorded in months. Offspring height was measured without shoes to the nearest 0.1 cm using a Harpenden stadiometer. A Lunar prodigy narrow fan beam densitometer was used to perform a whole body DXA scan from which fat mass was measured as described previously [24].

### Statistical analysis

Analysis was conducted using Stata version 12.0 MP2 (Stata Inc., TX, USA).

The distributions of shear velocity, liver volume, AST, AGT, GGT and haptoglobin were positively skewed but with a natural log transformation were approximately normally distributed. We use medians and interquartile range to describe these variables and natural logged values in multivariable models. Birthweight was standardized by gestational age in completed weeks, using the cohort gestational age distribution.

A series of multivariable regression models were constructed in order to examine the associations between maternal characteristics and offspring outcomes and to explore the impact of adjustment for potential confounding and mediating factors. For USS-determined fatty liver results are presented as odds ratio and 95 % confidence intervals and for liver volume, shear velocity, ALT, AST, GGT and haptoglobin results are presented as ratio of geometric means and 95 % confidence intervals and were obtained by back transforming coefficients.

Maternal-offspring pairs were included in the multivariable analyses if they had complete data on all variables used in any model. Therefore numbers vary between USS outcomes and blood-based outcomes but are the same across models for each outcome (*N* = 1 215 and *N* = 2 359 respectively). Confounders and/or mediators to be included in the models were decided upon a priori. We considered the following potential confounders: maternal age at delivery, parity, maternal pre-pregnancy alcohol intake, household social class and maternal pre-pregnancy BMI. Potential mediators were birthweight and offspring concurrent fat mass.

Whilst offspring measures of lipid and glucose metabolism are closely associated with NAFLD as previously shown in this population [20], they cannot be confounders here as the exposure of interest (i.e. maternal diabetes/glycosuria) precedes these. Moreover, as the direction of their relationship with NAFLD (cause, consequence or both) remains unclear, adjusting for these may constitute adjusting for a common effect which would bias results [25].

In the basic model (model 1) we controlled for offspring age at the time of outcome assessment and gender. In the confounder adjusted model (model 2) we additionally adjusted for the potential confounding by maternal age at delivery, parity, maternal pre-pregnancy alcohol intake, household social class, and when considering pregnancy diabetes/glycosuria as the exposure we additionally adjusted for maternal pre-pregnancy BMI. In model 3 we also adjusted for possible mediation by birthweight for gestational age and in model 4 we considered mediation by offspring’s concurrent adiposity by adjusting for DXA assessed fat mass, height and height squared (assessed at the 17–18 year clinic). In sensitivity analyses, we examined whether adjustment for offspring concurrent BMI instead of DXA-assessed fat mass altered the associations examined (*N* = 1 215 for USS outcomes and 2 358 for blood-based outcomes).

## Results

Table 1 shows the characteristics of mother-offspring pairs included in the analysis of either USS or blood-based outcomes, compared to mother-offspring pairs who are not included in either analyses but who attended the 17–18 year clinic, and to cohort members who did not attend the 17–18 year clinic. Of the 2 753 mother-offspring pairs who were included in either analyses of USS (*N* = 1 215) or blood-based outcomes (*N* = 2,359), 18.9 % of the mothers were overweight or obese and 4.1 % of mothers had pregnancy diabetes or glycosuria. Of the 1 215 offspring included in analyses, 2.1 % (*N* = 25) had USS fatty liver. Mothers of offspring who attended the 17–18 year clinic but were not included in either analyses (mainly due to missing outcome data) were younger, consumed less alcohol before pregnancy and were more likely to be from a lower social class household compared to those who were included in analyses. Offspring who attended the 17–18 year clinic but were not included in analyses had a lower mean birthweight and greater mean fat mass compared to those offspring who were included in analyses. Mothers of offspring who did not attend the 17–18 year clinic had a higher pre-pregnancy BMI, were younger, had more previous pregnancies, consumed less alcohol during pregnancy and were more likely to be from a manual social class household compared to the mothers of offspring who were included in either analyses. Offspring who did not attend clinic at age 17–18 years were more likely to be male and had a lower mean birthweight compared to those included in either USS or blood-based analyses (Table 1).

**Table 1:** Comparison of maternal and offspring characteristics of participants who were included in either the USS based outcomes and/or blood-based outcome analyses, to participants not included in either analyses but attended clinic at age 17–18 years and to those who did not attend the clinic at age 17–18 years

Characteristic	Included in either analysis of blood-based or USS outcomes (reference group)		Attended 17–18 year clinic but not included in either analysis			Did not attend 17–18 year clinic		
	N	Mean (SD) or median (IQR) or N (%) as indicated	N	Mean (SD) or median (IQR) or N (%) as indicated	*P* value*	N	Mean (SD) or median (IQR) or N (%) as indicated	*P* value*
Maternal								
Pre-pregnancy BMI (mean, SD)	2 753	22.8 (3.7)	1 520	23.0 (3.7)	0.15	6,940	23.0 (4.0)	0.01
Pre-pregnancy BMI (N, %)	2 753		1 520		0.15	6,940		0.004
Underweight/Normal		2 234 (81.2)		1 206 (79.3)			5,451 (78.4)	
Overweight/Obese		519 (18.9)		314 (20.7)			1,489 (21.5)	
Diabetes status (N, %)	2 753		1 770		0.98	7,480		0.93
No diabetes or glycosuria		2 639 (95.9)		1 697 (95.9)			7 173 (95.9)	
Existing diabetes/Gestational diabetes/Glycosuria		114 (4.1)		73 (4.1)			307 (4.1)	
Maternal age (years) (mean, SD)	2 753	29.5 (4.5)	1 953	28.8 (4.7)	<0.001	8,864	27.3 (5.0)	<0.001
Parity (N, %)	2 753		1 797		0.99	8,003		<0.001
0		1 326 (48.2)		866 (48.2)			3 444 (43.0)	
1		988 (35.9)		591 (32.9)			2 801 (35.0)	
2+		439 (16.0)		340 (18.9)			1 758 (22.0)	
Pre pregnancy alcohol intake (N, %)	2 753		s		<0.001	8,071		<0.001
<1 glass per week		1 141 (41.5)		863 (46.8)			3 833 (47.5)	
1+ glass per week		1 612 (58.6)		980 (53.2)			4 238 (52.5)	
Family social class (N, %)	2 753		1 594		<0.001	6,829		<0.001
Non-manual		2 432 (88.3)		1 344 (84.3)			5 228 (76.6)	
Manual		321 (11.7)		250 (15.7)			1 601 (23.4)	
Offspring								
Age (months) (mean, SD)	2 753	213.5 (4.7)	1 864	213.5 (5.3)	0.97			
Male (N, %)	2 753	1 300 (47.2)	1 953	676 (39.3)	<0.001	8,862	4 938 (55.7)	<0.001
Gestational age (weeks) (mean, SD)	2 753	39.5 (1.7)	1 953	39.5 (1.9)	0.42	8,864	39.5 (1.9)	0.17
Birthweight (g) (mean, SD)	2 753	3449.6 (514.1)	1 900	3400.0 (538.7)	0.002	8,746	3406.2 (546.7)	<0.001
Fat mass (g) (mean, SD)	2 753	17462.3 (9836.2)	1 670	19661.2 (11434.4)	<0.001			
Ultrasound fatty liver (N, %)	1 215	25 (2.1)	381	11 (2.9)	0.34			
Ultrasound liver volume (cm^3^) (median, IQR)	1 215	1581.1 (1305.8, 1856.5)	450	1588.6 (1302.1, 1875.9)	0.94			
Ultrasound shear velocity (m/sec) (median, IQR)	1 215	1.2 (1.1, 1.3)	382	1.3 (1.1, 1.3)	0.05			
ALT (U/l) (median, IQR)	2 359	15.0 (12.0, 19.5)	614	15.2 (11.7, 19.7)	0.60			
AST (U/l) (median, IQR)	2 359	19.6 (16.8, 23.2)	614	19.6 (16.6, 23.3)	0.35			
GGT (U/l) (median, IQR)	2 359	16.0 (13.0, 21.0)	614	16.0 (13.0, 21.0)	0.63			
Haptoglobin (g/l) (median, IQR)	2 359	1.0 (0.7, 1.3)	608	1.0 (0.7, 1.3)	0.41			
Glucose (mmol/l) (median, IQR)	2 350	5.0 (4.8, 5.2)	651	5.0 (4.8, 5.3)				
Insulin (pmol/l) (median, IQR)	2 322	39.6 (29.0, 55.7)	628	43.6 (31.0, 59.2)				
Triglycerides (mmol/l) (median, IQR)	2 350	0.8 (0.6, 1.0)	651	0.8 (0.6, 1.0)				
Total cholesterol (median, IQR)	2 350	3.7 (3.3, 4.2)	651	3.7 (3.2, 4.2)				
LDLc (mmol/l) (median, IQR)	2 350	0.3 (0.3, 0.5)	651	0.3 (0.3, 0.4)				
HDLc (mmol/l) (median, IQR)	2 350	1.2 (1.1, 1.4)	651	1.2 (1.1, 1.5)				

*USS* Ultrasound scan, *ALT* alanine amino transferase, *AST* aspartate amino transferase, *GGT* gamma- glutamyltransferase, *LDLc* low density lipoprotein cholesterol, *HDLc* high density lipoprotein cholesterol, *IQR* Interquartile range, *SD* standard deviation

*P-value for the null hypothesis of no difference compared to those included in either analysis of blood based or USS outcomes group (reference group)

Table 2 shows the multivariable associations of maternal pregnancy diabetes/glycosuria with offspring outcomes. Offspring whose mothers had pregnancy diabetes/glycosuria were more likely to have USS fatty liver and had a higher mean shear velocity in the age and gender adjusted model (model 1). Adjustment for potential confounding factors (including maternal pre-pregnancy BMI), did not notably alter these associations. Further adjustment for potential mediation by offspring birthweight (model 3) or later offspring adiposity (model 4) did not markedly change the magnitude of these associations. In the confounder and mediator adjusted model, exposure to diabetes/glycosuria in utero was associated with more than a 6-fold increase in the risk of having a fatty liver compared to offspring of mothers with no pregnancy diabetes/glycosuria during pregnancy. There was no evidence of an association for maternal pregnancy diabetes/glycosuria with liver volume, fasting blood ALT, AST, GGT or haptoglobin in any of the multivariable models.

**Table 2:** Multivariable associations of maternal diabetes/glycosuria with offspring USS and blood-based markers of non-alcoholic fatty liver disease (*N* = 1 215 or 2 359 as indicated)

	No diabetes or glycosuria (reference)	Diabetes/glycosuria	*P* value
USS fatty liver			
N	18/1 153^a^	7/62^a^	
Model 1	1	9.07 (3.53, 23.30)	<0.001
Model 2	1	6.74 (2.47, 18.40)	<0.001
Model 3	1	6.57 (2.38, 18.10)	<0.001
Model 4	1	6.72 (1.89, 24.00)	0.003
USS liver volume			
N	1 153	62	
Model 1	1	1.04 (0.98, 1.11)	0.20
Model 2	1	1.03 (0.96, 1.09)	0.45
Model 3	1	1.02 (0.96, 1.08)	0.57
Model 4	1	1.01 (0.95, 1.08)	0.70
USS shear velocity			
N	1 153	62	
Model 1	1	1.11 (1.06, 1.16)	<0.001
Model 2	1	1.10 (1.05, 1.15)	<0.001
Model 3	1	1.09 (1.05, 1.15)	<0.001
Model 4	1	1.08 (1.04, 1.13)	0.001
ALT			
N	2 270	89	
Model 1	1	1.04 (0.95, 1.14)	0.40
Model 2	1	1.03 (0.94, 1.13)	0.50
Model 3	1	1.04 (0.94, 1.13)	0.44
Model 4	1	1.01 (0.93, 1.11)	0.76
AST			
N	2 270	89	
Model 1	1	1.03 (0.97, 1.09)	0.38
Model 2	1	1.03 (0.97, 1.09)	0.32
Model 3	1	1.03 (0.98, 1.10)	0.27
Model 4	1	1.03 (0.97, 1.09)	0.37
GGT			
N	2 270	89	
Model 1	1	1.03 (0.96, 1.11)	0.43
Model 2	1	1.03 (0.95, 1.11)	0.50
Model 3	1	1.03 (0.96, 1.11)	0.43
Model 4	1	1.01 (0.94, 1.09)	0.77
Haptoglobin			
N	2 270	89	
Model 1	1	0.98, (0.87, 1.10)	0.73
Model 2	1	0.97 (0.86, 1.09)	0.57
Model 3	1	0.97 (0.86, 1.09)	0.62
Model 4	1	0.95 (0.84, 1.07)	0.42

*USS* Ultrasound scan, *ALT* alanine amino transferase, *AST* aspartate amino transferase, *GGT* gamma- glutamyltransferase

^a^numerator represents the number of offspring with USS fatty liver

Model 1: adjusted for offspring age at outcome assessment and gender

Model 2: (confounder adjusted) as model 1 plus additional adjustment for maternal age, parity, maternal alcohol intake, highest household manual social class and maternal pre-pregnancy BMI

Model 3: (confounder and mediator adjusted) as model 2 plus additional adjustment for birthweight for gestational age (z scores)

Model 4: (confounder and mediator adjusted) as model 2 plus additional adjustment for DXA-assessed fat mass, height and height squared

All estimates are geometric means (95%CI) except for the estimate for USS fatty liver (yes/no) which is an OR (95%CI)

Table 3 shows the multivariable associations of maternal pre-pregnancy obesity and BMI with offspring outcomes. Offspring of overweight/obese mothers were more than twice as likely to have USS fatty liver, and had greater liver volume and higher shear velocity in the basic and confounder adjusted models (models 1 and 2) compared to offspring of underweight/normal weight mothers. This association remained when offspring birthweight was added to the model as a potential mediator (model 3), whilst associations were attenuated to the null when adjusting for offspring concurrent fat mass (model 4). The association of maternal overweight/obesity with liver volume was attenuated in both mediator adjusted models (model 3 and 4). There was no strong evidence of an association of maternal pre-pregnancy overweight/obese with ALT, AST, GGT or haptoglobin in any of the multivariable models. Similar patterns of association were seen when considering maternal pre-pregnancy BMI as a continuous exposure (per SD change in pre-pregnancy BMI).

**Table 3:** Multivariable associations of maternal pre-pregnancy obesity status and BMI with offspring USS and blood-based markers of non-alcoholic fatty liver disease (*N* = 1 215 or 2 359 as indicated)

	Pre-pregnancy BMI category			Per SD of BMI	
	Underweight/normal (reference category)	Overweight/obese	*P* value		*P* value
USS fatty liver					
N	15/966^a^	10/249^a^		25/1 215^a^	
	Odds ratio (95 % confidence intervals)			Odds ratio (95 % confidence intervals)	
Model 1	1	2.72 (1.20, 6.15)	0.02	1.66 (1.27, 2.17)	<0.001
Model 2	1	2.70 (1.18, 6.17)	0.02	1.68 (1.28, 2.21)	<0.001
Model 3	1	2.45 (1.06, 5.99)	0.04	1.63 (1.23, 2.17)	0.001
Model 4	1	0.36 (0.11, 1.14)	0.08	0.86 (0.58, 1.28)	0.46
USS liver volume					
N	966	249		1 215	
	Ratio of geometric means (95 % confidence intervals)			Ratio of geometric means (95 % confidence intervals)	
Model 1	1	1.03 (1.00, 1.07)	0.01	1.03 (1.02, 1.04)	<0.001
Model 2	1	1.03 (1.00, 1.07)	0.02	1.03 (1.02, 1.05)	<0.001
Model 3	1	1.01 (0.98, 1.05)	0.04	1.02 (1.00, 1.04)	0.001
Model 4	1	0.98 (0.95, 1.02)	0.75	1.01 (1.00, 1.02)	0.19
USS shear velocity					
N	966	249		1 215	
	Ratio of geometric means (95 % confidence intervals)			Ratio of geometric means (95 % confidence intervals)	
Model 1	1	1.03 (1.01, 1.06)	0.08	1.02 (1.01, 1.03)	<0.001
Model 2	1	1.03 (1.01, 1.06)	0.08	1.02 (1.01, 1.03)	0.001
Model 3	1	1.03 (1.00, 1.05)	0.38	1.02 (1.00, 1.03)	0.002
Model 4	1	1.00 (0.98, 1.03)	0.46	1.00 (0.99, 1.02)	0.37
ALT					
N	1 921	438		2 359	
	Ratio of geometric means (95 % confidence intervals)			Ratio of geometric means (95 % confidence intervals)	
Model 1	1	1.03 (0.98, 1.08)	0.21	1.02 (1.00, 1.04)	0.06
Model 2	1	1.03 (0.98, 1.07)	0.25	1.01 (0.99, 1.04)	0.35
Model 3	1	1.03 (0.99, 1.08)	0.19	1.02 (1.00, 1.04)	0.04
Model 4	1	0.95 (0.91, 1.00)	0.04	0.98 (0.96, 1.00)	0.05
AST					
N	1 921	438	2,359	2 359	
	Ratio of geometric means (95 % confidence intervals)			Ratio of geometric means (95 % confidence intervals)	
Model 1	1	0.99 (0.96, 1.02)	0.550	1.00 (0.99, 1.01)	0.54
Model 2	1	0.99 (0.96, 1.02)	0.45	0.99 (0.97, 1.01)	0.32
Model 3	1	0.99 (0.96, 1.02)	0.60	1.00 (0.99, 1.01)	0.72
Model 4	1	0.97 (0.95, 1.00)	0.07	0.99 (0.98, 1.00)	0.05
GGT					
N	1 921	438	2,359	2 359	
	Ratio of geometric means (95 % confidence intervals)			Ratio of geometric means (95 % confidence intervals)	
Model 1	1	1.02 (0.98, 1.05)	0.39	1.01 (0.99, 1.02)	0.26
Model 2	1	1.02 (0.98, 1.05)	0.38	1.00 (0.97, 1.02)	0.74
Model 3	1	1.02 (0.98, 1.06)	0.29	1.01 (1.00, 1.03)	0.16
Model 4	1	0.95 (0.92, 0.99)	0.01	0.98 (0.97, 0.99)	0.01
Haptoglobin					
N	1 921	438		2 359	
	Ratio of geometric means (95 % confidence intervals)			Ratio of geometric means (95 % confidence intervals)	
Model 1	1	1.03 (0.98, 1.10)	0.96	1.02 (1.00, 1.05)	0.04
Model 2	1	1.03 (0.97, 1.09)	0.97	1.00 (0.97, 1.04)	0.79
Model 3	1	1.03 (0.97, 1.10)	0.30	1.02 (1.00, 1.05)	0.05
Model 4	1	0.96 (0.90, 1.02)	0.18	0.99 (0.97, 1.01)	0.41

*USS* Ultrasound scan, *ALT* alanine amino transferase, *AST* aspartate amino transferase, *GGT* gamma- glutamyltransferase

^a^numerator represents the number of offspring with USS fatty liver

Model 1: adjusted for offspring age at outcome assessment and gender

Model 2: (confounder adjusted) as model 1 plus additional adjustment for maternal age, parity, maternal alcohol intake, highest household manual social class

Model 3: (confounder and mediator adjusted) as model 2 plus additional adjustment for birthweight for gestational age

Model 4: (confounder and mediator adjusted) as model 2 plus additional adjustment for DXA-assessed fat mass, height and height squared

Additional file 1: Table S1 shows the distribution of outcomes across the four categories of maternal pre-existing diabetes, gestational diabetes, glycosuria and no maternal diabetes/glycosuria and the univariable associations between exposures and outcomes. Associations were generally consistent for these three categories suggesting that it was appropriate to combine them in the main analysis. Additional file 1: Tables S2 and S3 show the multivariate model results (model 4) for the assocaition of maternal diabetes/glycosuria and pre-pregnancy obesity with USS determined fatty liver, respectively.

There were no notable differences in the associations of maternal pregnancy diabetes/glycosuria and maternal pre-pregnancy BMI with offspring outcomes when offspring concurrent BMI was used as a measure of offspring adiposity, compared to using DXA-assessed fat mass (model 4) (see Additional file 1: Tables S4 and S5).

## Discussion

In this general population prospective birth cohort, we used USS to determine liver pathologies including fatty liver, estimated liver volume and shear velocity (a marker of liver stiffness or fibrosis). There were no known cases of liver disease in this cohort and we removed the small number of participants who had reported continuous harmful drinking in the previous two years. Therefore it is reasonable that in our cohort USS fatty liver is likely to represent NAFLD.

In our study, maternal pregnancy diabetes/glycosuria was positively associated with a 6-fold increase in odds of offspring NAFLD and higher shear velocity at mean age 17.8 years even when adjusting for maternal pre-pregnancy BMI. Offspring birthweight or concurrent adiposity did not appear to mediate this association. Maternal overweight/obesity and pre-pregnancy BMI across the BMI distribution were associated with greater odds of NAFLD, higher liver volume and greater shear velocity (a measure of liver fibrosis) in adolescent offspring, even when adjusting for potential confounders. For these associations too, birthweight did not appear to be an important mediator. In contrast, adjusting for offspring concurrent adiposity attenuated these associations, suggesting that offspring’s own adiposity mediates associations between maternal pre-pregnancy adiposity and offspring liver outcomes.

Little is known about the early life determinants of NAFLD as it is a relatively newly recognised condition and therefore has not commonly been assessed in large epidemiological studies. Our study extends findings from two small studies; one that reported greater IHCL content in infants of obese mothers with diabetes compared to infants of non-obese non-diabetic mothers and the other study reported a positive association between maternal pre-pregnancy BMI and infants’ IHCL content [15, 16]. These studies did not examine the association of maternal BMI and pregnancy diabetes separately as we have done here. Furthermore, here NAFLD was assessed much later on in the life course, which is important in terms of understanding any lasting effect that could result in important adverse health outcomes.

The results presented in this paper add to the growing body of evidence suggesting that maternal pregnancy diabetes is associated with later offspring cardiometabolic health. Maternal pregnancy diabetes has been associated with higher risk of offspring adiposity [3, 26], type 2 diabetes risk [27] and higher fasting glucose and insulin in adolescence [8]. Our results suggest that the risk of offspring NAFLD is also elevated. Sibling comparisons, in which siblings exposed to pregnancy diabetes are compared to their siblings not exposed to pregnancy diabetes, thus inherently controlling for measured and unmeasured shared genetic and familial environment, provide convincing causal evidence that associations of maternal pregnancy diabetes with offspring greater fatness can be attributed to a direct intrauterine mechanism [26, 27]. Whilst we are unable to confidently infer that associations observed here are causal, it is reasonable to assume that similar intrauterine mechanisms are driving the association with NAFLD reported here.

It has been estimated that up to half of gestational diabetes cases are attributable to pre-pregnancy overweight and obesity [28] and in a systematic review and meta-analysis it was calculated that for one unit increase in pre-pregnancy BMI the risk of gestational diabetes increased by 0.92 % (95 % CI: 0.73,1.10 %) [29]. Here, the association of maternal pregnancy diabetes/glycosuria and NAFLD remained even after adjusting for potential confounding by maternal pre-pregnancy BMI and after accounting for birthweight and offspring concurrent adiposity. Similarly, adjusting for maternal early pregnancy BMI in the aforementioned sibling study did not account for the higher BMI of young men exposed to diabetes in utero compared to their unexposed brothers [26]. This suggests that familial adiposity, whether due to shared genetics or behaviour, is not a main or only driver of the association between maternal diabetes/glycosuria in pregnancy and offspring NAFLD; and that maternal pregnancy diabetes/glycosuria and offspring NAFLD are associated through other mechanisms. One such mechanism may be greater offspring insulin resistance, though the temporal relationship between liver fat accumulation and insulin resistance, both hepatic and peripheral, is yet to be clarified, and for this reason we did not adjust for measures of lipid and glucose metabolism in our analysis. Other potential mechanisms include epigenetic differences (e.g. differential DNA methylation) [30] and differences in offspring gut microbiota [31].

Authors of two recent systematic reviews [32, 3] concluded that the evidence supporting association between gestational diabetes [32], pregnancy diabetes [3] and greater offspring adiposity (which is strongly associated with NAFLD) remains inconclusive due to the attenuation of the association when adjusting for maternal pre-pregnancy BMI. However, most included studies were conducted in settings with no universal screening for gestational diabetes, with diagnostic tests only being offered to those at greater risk, namely overweight/obese [1]. This would then result in over-attenuation of the maternal diabetes and offspring outcome association when adjusting for maternal pregnancy BMI, as has previously been demonstrated [1].

In contrast, the association of maternal pre-pregnancy BMI with NAFLD was attenuated to the null upon adjustment for offspring adiposity. This suggests that familial characteristics (genetic or behavioural) related to family adiposity underlie the association between maternal pre-pregnancy BMI and offspring NAFLD as opposed to a direct intrauterine effect. These findings are also consistent with the sibling comparison study in which no association between maternal early pregnancy BMI and offspring BMI at 18 years was noted when only brothers were compared, suggesting that the positive association between maternal and offspring BMI observed in the overall population and in other studies may be due to confounding by shared behaviour, environmental exposures or genes inherently controlled for in the sibling comparison [33].

We found modest associations of maternal pre-pregnancy BMI and pregnancy diabetes/glycosuria with blood-based outcomes. These were broadly in agreement with results for the USS-determined outcomes. In a separate publication, based on this cohort, we have shown that those participants with NAFLD had higher ALT, AST, GGT and haptoglobin. That said, the relatively modest associations found here are likely a result of the liver enzymes and haptoglobin being much cruder measures of liver fat and indeed of liver health than USS based measures [34, 35]. USS is considered superior to liver enzyme for the diagnosis of NAFLD which is why we considered the USS measures to be our primary outcomes from the outset. Despite its shortcomings compared to other imaging modalities such as MRI and CT scans, USS is the most feasible and commonly used method for assessing liver fat in large scale epidemiological studies such as ALSPAC.

Our study has limitations. Loss to follow up is common feature of many prospective cohort studies including ours. Although loss to follow up is an issue in terms of reduction of statistical power, it is unlikely to have biased our results as this loss to follow up would be non-differential i.e. offspring would be unaware of their outcomes (participants would have to know if they had NAFLD).

Information on existing maternal diabetes and pre-pregnancy weight, height and alcohol consumption were collected by self-reported questionnaire. This non-differential misclassification would result in an underestimate of associations of interest. However, as mentioned earlier maternal self-reported weight was highly correlated with maternal weight measured at the first antenatal clinical visit (*r* = 0.95), which provides confidence in the self-reported measures.

Due to the small numbers of women with existing diabetes, gestational diabetes and glycosuria these groups were combined for the analysis. The prevalence of gestational diabetes in our cohort was low, likely due to the lack of universal screening and it is possible that women with glycosuria are a mixture of women with undiagnosed gestational diabetes and women with high circulating glucose levels but below the threshold that would be used to diagnose gestational diabetes. We cannot rule out renal glycosuria though the prevalence of this condition is low. Inclusion of women with renal glycosuria or false positive dip stick tests would result in an underestimate of the association of interest. Lastly, we have previously reported that glycosuria is related to macrosomia in this cohort [7], and with cardiovascular death in a US study, [36] giving face validity to its use as an assessment of hyperglycaemia.

Despite the large number of participants in our study the numbers with diagnosed gestational diabetes was low and we lacked information on maternal glucose tolerance and therefore larger studies with better measures of maternal glucose tolerance are required. Different study methods such as sibling comparison studies (such as those conducted by Dabelea et al. [27] and Lawlor et al. [26]) may help to determine whether these associations are at least in part driven by intrauterine mechanisms or are fully explained by shared familial genetic or environmental characteristics.

USS is not the ‘gold standard’ for identifying NAFLD; however, it is neither feasible nor ethical to undertake liver biopsies in a large cohort of healthy people. Studies have shown USS to accurately identify moderate to severe steatosis compared with liver biopsy in adults and children [37, 38] thus, our prevalence estimate for NAFLD may reflect the moderate to severe end of the spectrum of this disease. The small number of offspring with fatty liver reflects the relatively young age. The ARFI measure of liver fibrosis used in our study is a relatively new measure, but it has been validated in a small number of clinical studies [39, 40]. Importantly, we have looked at a range of outcomes, including blood-based and those measured by USS, which can be considered to be indicators of NAFLD or its liver complications, which counters some of the limitations of not having gold standard biopsy outcomes. Even though this is the largest study to date assessing the association of maternal pregnancy BMI and diabetes/glycosuria with NAFLD in an adolescent population; the number of offspring with NAFLD was small though on the lower end of range compared to a similar population [41].

## Conclusions

In summary, our study adds to the small body of evidence of the early life origins of NAFLD and suggests that maternal pregnancy diabetes/glycosuria is associated with greater risk of offspring NAFLD and higher liver shear velocity (a marker of liver stiffness) even when accounting for potential mediation by offspring concurrent adiposity. Conversely, maternal pre-pregnancy BMI was no longer associated with offspring NAFLD when accounting for offspring adiposity. Results suggest that maternal pregnancy diabetes/glycosuria is associated with offspring NAFLD through mechanisms other than offspring’s own adiposity. These results add to the literature linking NAFLD and diabetes and demonstrate that the two may share common antecedents. Results also suggest that maternal pregnancy diabetes/glycosuria is associated with offspring NAFLD through mechanisms other than offspring’s own adiposity. Finally, this study adds to the growing body of evidence demonstrating the importance of maternal health in pregnancy and its associations with long term health in the next generation.

## Additional file

Additional file 1: Table S1. Univariable associations of maternal diabetes status, by maternal existing diabetes, gestational diabetes and glycosuria compared to no diabetes/glycosuria with offspring USS and blood-based markers of non-alcoholic fatty liver disease. **Table S2.** Results of the multivariable model (model 4) of the association of maternal diabetes/glycosuria with offspring USS determined fatty liver. **Table S3.** Results of the multivariable model (model 4) of the association of maternal pre-pregnancy obesity status and BMI with offspring USS determined fatty liver. **Table S4. ** Multivariable associations (model 4, with adjustment for offspring concurrent BMI) of maternal diabetes/glycosuria with offspring USS and blood-based markers of non-alcoholic fatty liver disease. (*N* = 1,215 or 2,358 as indicated). **Table S5. ** Multivariable associations of maternal pre-pregnancy BMI ((model 4, with adjustment for offspring concurrent BMI)) with offspring USS and blood-based markers of non-alcoholic fatty liver disease. (*N* = 1,215 or 2,358 as indicated). (DOCX 26 kb)

## Abbreviations

ALSPAC: Avon Longitudinal Study of Parents and Children
ALT: amino transferase
AST: aspartate amino transferase
BMI: bmody mass index
GGT: gamma- glutamyltransferase
NAFLD: non-alcoholic fatty liver disease
USS: ultrasonography scan
