# Supplementary material for: Association of maternal diabetes/glycosuria and pre-pregnancy body mass index with offspring indicators of non-alcoholic fatty liver disease
Source: BMC Pediatr. 2016 Mar 31;16:47. doi: 10.1186/s12887-016-0585-y (PMC4818433; doi:10.1186/s12887-016-0585-y)
Supplement: Additional file 1: Table S1. — Univariable associations of maternal diabetes status, by maternal existing diabetes, gestational diabetes and glycosuria compared to no diabetes/glycosuria with offspring USS and blood-based markers of non-alcoholic fatty liver disease. Table S2. Results of the multivariable model (model 4) of the association of maternal diabetes/glycosuria with offspring USS determined fatty liver. Table S3. Results of the multivariable model (model 4) of the association of maternal pre-pregnancy obesity status and BMI with offspring USS determined fatty liver. Table S4. Multivariable associations (model 4, with adjustment for offspring concurrent BMI) of maternal diabetes/glycosuria with offspring USS and blood-based markers of non-alcoholic fatty liver disease. (N = 1,215 or 2,358 as indicated). Table S5. Multivariable associations of maternal pre-pregnancy BMI ((model 4, with adjustment for offspring concurrent BMI)) with offspring USS and blood-based markers of non-alcoholic fatty liver disease. (N = 1,215 or 2,358 as indicated). (DOCX 26 kb) [file 12887_2016_585_MOESM1_ESM.docx]

**Table S1: Univariable associations of maternal diabetes status, by maternal existing diabetes, gestational diabetes and glycosuria compared to no diabetes/ glycosuria with offspring USS and blood-based markers of non-alcoholic fatty liver disease**

|  | **No diabetes / glycosuria** | **Existing diabetes** | **Gestational diabetes** | **Glycosuria** | P value  (3 d.f.)^a^ |
| --- | --- | --- | --- | --- | --- |
|  | N=1,153 | N=7 | N=8 | N=47 | - |
| Ultrasound fatty liver  (N, %) | 18 (1.6) | 1 (14.3) | 1 (12.5) | 5 (10.6) | - |
| Odds ratio (95% CI) | 1 | 10.5 (1.2, 91.9) | 9.0 (1.1, 77.1) | 7.5 (2.7, 21.2) | 0.002 |
|  | N=1,153 | N=7 | N=8 | N=47 |  |
| Ultrasound liver volume (cm^3^) (mean, SD) | 1577.2 (1305.7, 1848.5) | 1445.6 (1196.3, 1935.8) | 1575.4 (1186.8, 2068.7) | 1630.1 (1344.9, 1920.3) |  |
| Ratio of geometric means (95% CI) | 1 | 1.0 (0.8, 1.2) | 1.0 (0.8, 1.2) | 1.0 (0.5, 1.1) | 1.0 |
|  | N=1,153 | N=7 | N=8 | N=47 | - |
| Ultrasound shear velocity (m/sec) (median, IQR) | 1.2 (1.1, 1.3) | 1.5 (1.0, 1.8) | 1.5 (1.0, 1.6) | 1.3 (1.1, 1.4) | - |
| Ratio of geometric means (95% CI) | 1 | 1.2 (1.1, 1.4) | 1.1 (1.0, 1.3) | 1.1 (1.0, 1.1) | <0.001 |
|  | N=2,270 | N=10 | N=12 | N=67 | - |
| ALT (U/l) (median, IQR) | 15.0 (12.0, 19.5) | 13.6 (11.3, 19.9) | 13.9 (11.4, 19.9) | 15.7 (13.0, 18.8) |  |
| Ratio of geometric means (95% CI) | 1 | 0.9 (0.7, 1.2) | 0.9 (0.7, 1.2) | 1.0 (0.9, 1.2) | 0.74 |
|  | N=2,270 | N=10 | N=12 | N=67 |  |
| AST (U/l) (median, IQR) | 19.6 (16.8, 23.2) | 20.3 (17.5, 21.6) | 18.9 (15.6, 22.3) | 19.6 (17.0, 24.4) | - |
| Ratio of geometric means (95% CI) | 1 | 1.0 (0.9, 1.2) | 0.9 (0.8, 1.1) | 1.0 (1.0, 1.1) | 0.52 |
|  | N=2,270 | N=10 | N=12 | N=67 |  |
| GGT (U/l) (median, IQR) | 16.0 (13.0, 21.0) | 20.5 (14.0, 27.0) | 16.0 (11.5, 20.0) | 17.0 (14.0, 21.0) | - |
| Ratio of geometric means (95% CI) | 1 | 1.2 (1.0, 1.5) | 0.9 (0.7, 1.0) | 1.0 (0.9, 1.1) | 0.17 |
|  | N=2,270 | N=10 | N=12 | N=67 |  |
| Haptoglobin (g/l) (median, IQR) | 1.0 (0.7, 1.3) | 1.0 (0.5, 1.4) | 1.1 (0.6, 1.6) | 1.0 (0.8, 1.3) | - |
| Ratio of geometric means (95% CI) | 1 | 0.9 (0.7, 1.3) | 1.0 (0.7, 1.4) | 1.0 (0.9, 1.1) | 1.0 |

USS: Ultrasound scan; ALT: alanine amino transferase; AST: aspartate amino transferase; GGT: gamma- glutamyltransferase;

IQR: Interquartile range; SD: standard deviation; CI: confidence intervals

^a^: P value for the null hypothesis of no difference between the four groups (i.e. 3 degrees of freedom)

**Table S2: Results of the multivariable model (model 4) of the association of maternal diabetes/glycosuria with offspring USS determined fatty liver**

| **Variable** | **OR and 95% confidence intervals** | **P value** |
| --- | --- | --- |
| Maternal diabetes/glycosuria (yes vs.no) | 6.72 (1.89, 24.00) | 0.003 |
| Gender (male vs. female) | 0.24 (0.06, 0.99) | 0.05 |
| Offspring age in months | 1.06 (0.97, 1.16) | 0.22 |
| Maternal age at delivery in years | 1.01 (0.90, 1.14) | 0.83 |
| Parity  0  1  2+ | 1  1.61 (0.53, 4.93)  2.31 (0.52, 10.14) | 0.40  0.27 |
| Maternal alcohol consumption during pregnancy (1+ glass vs. week  <1 glass/ week ) | 0.92 (0.34, 2.48) | 0.88 |
| Household social class (manual vs. non-manual social class) | 0.83 (0.20, 3.48) | 0.80 |
| Maternal pre pregnancy BMI (kg/m^2^) | 0.93 (0.83, 1.04) | 0.20 |
| Fat mass (grams) | 1.00 (1.00, 1.00) | <0.001 |
| Height (cm) | 2.17 (0.34, 13.95) | 0.42 |
| Height squared | 1.00 (0.99, 1.00) | 0.41 |

**USS- ultrasound scan; OR; odds ratio**

**Table S3: Results of the multivariable model (model 4) of the association of maternal pre-pregnancy obesity status and BMI with offspring USS determined fatty liver**

|  | **Pre-pregnancy BMI category** | | **Per SD of pre-pregnancy BMI** | |
| --- | --- | --- | --- | --- |
|  | **OR and 95% confidence intervals** | **P value** | **OR and 95% confidence intervals** | **P value** |
| Maternal overweight/obesity (yes/no) | 0.36 (0.11, 1.14) | 0.08 |  |  |
| Maternal pre-pregnancy BMI (per SD) |  |  | 0.86 (0.58, 1.28) | 0.46 |
| Gender (male vs. Female) | 0.25 (0.06, 0.99) | 0.05 | 0.25 (0.06, 0.99) | 0.05 |
| Offspring age (months) | 1.06 (0.97, 1.15) | 0.53 | 1.05 (0.96, 1.15) | 0.27 |
| Maternal age at delivery (years) | 1.04 (0.93, 1.15) | 0.53 | 1.03 (0.92, 1.15) | 0.60 |
| Parity  0  1  2+ | 1  1.43 (0.49, 4.22)  1.65 (0.39, 6.95) | 0.51  0.49 | 1  1.35 (0.46, 3.97)  1.70 (0.41, 7.06) | 1  0.59  0.47 |
| Maternal alcohol consumption during pregnancy (1+ glass / week vs.  <1 glass/ week ) | 1.00 (0.38, 2.65) | 1.00 | 0.91 (0.35, 2.38) | 0.85 |
| Household social class (manual vs. non-manual social class) | 1.07 (0.29, 3.87) | 0.92 | 0.99 (0.27, 3.61) | 0.98 |
| Fat mass | 1.00 (1.00, 1.00) | <0.001 | 1.00 (1.00, 1.00) | <0.001 |
| Height (cm) | 2.21 (0.35, 13.91) | 0.40 | 1.96 (0.32, 11.91) | 0.46 |
| Height squared | 1.00 (0.99, 1.00) | 0.38 | 1.00 (0.99, 1.00) | 0.45 |

**USS- ultrasound scan; SD- standard deviation; OR; odds ratio**

**Table S4: Multivariable associations (model 4, with adjustment for offspring concurrent BMI) of maternal diabetes/glycosuria with offspring USS and blood-based markers of non-alcoholic fatty liver disease. (N=1,215 or 2,358 as indicated)**

|  | **No diabetes or glycosuria (reference)** | **Diabetes / glycosuria** |
| --- | --- | --- |
| **USS fatty liver** | | |
| N | 18 / 1,153^a^ | 7/ 62^a^ |
|  | Odds ratio (95% confidence intervals) | |
|  | 1 | 7.43 (2.05, 27.00) |
| **USS liver volume** | | |
| N | 1,153 | 62 |
|  | Ratio of geometric means (95% confidence intervals) | |
|  | 1 | 1.00 (0.94, 1.07) |
| **USS shear velocity** | | |
| N | 1,153 | 62 |
|  | Ratio of geometric means (95% confidence intervals) | |
|  | 1 | 1.09 (1.04, 1.14) |
| **ALT** | | |
| N | 2,269 | 89 |
|  | Ratio of geometric means (95% confidence intervals) | |
|  | 1 | 1.01 (0.93, 1.10) |
| **AST** | | |
| N | 2,269 | 89 |
|  | Ratio of geometric means (95% confidence intervals) | |
|  | 1 | 1.02 (0.97, 1.08) |
| **GGT** | | |
| N | 2,269 | 89 |
|  | Ratio of geometric means (95% confidence intervals) | |
|  | 1 | 1.01 (0.94, 1.09) |
| **Haptoglobin** | | |
| N | 2,269 | 89 |
|  | Ratio of geometric means (95% confidence intervals) | |
|  | 1 | 0.95 (0.85, 1.08) |

USS: Ultrasound scan; ALT: alanine amino transferase; AST: aspartate amino transferase; GGT: gamma- glutamyltransferase

^a^: numerator represents the number of offspring with USS fatty liver

Adjusted for offspring age at outcome assessment and gender, maternal age, parity, maternal alcohol intake, household manual social class, maternal pre-pregnancy BMI and offspring concurrent BMI

**Table S5: Multivariable associations of maternal pre-pregnancy BMI ((model 4, with adjustment for offspring concurrent BMI)) with offspring USS and blood-based markers of non-alcoholic fatty liver disease. (N=1,215 or 2,358 as indicated)**

|  | **Pre-pregnancy BMI category** | | **Per SD of BMI** |
| --- | --- | --- | --- |
|  | **Underweight / normal (reference category)** | **Overweight/ obese** |  |
| **USS fatty liver** | | | |
| N | 15/966^a^ | 10/ 249 ^a^ | 25/1,215 ^a^ |
|  | Odds ratio (95% confidence intervals) | | |
|  | 1 | 0.37 (0.12, 1.17) | 0.80 (0.53, 1.20) |
| **USS liver volume** | | | |
| N | 966 | 249 | 1,215 |
|  | Ratio of geometric means (95% confidence intervals) | | |
|  | 1 | 0.97 (0.93, 1.00) | 1.00 (0.98, 1.01) |
| **USS shear velocity** | | | |
| N | 966 | 249 | 1,215 |
|  | Ratio of geometric means (95% confidence intervals) | | |
|  |  | | |
|  | 1 | 1.00 (0.98, 1.03) | 1.00 (0.99, 1.01) |
| **ALT** | | | |
| N | 1,921 | 438 | 2,358 |
|  | Ratio of geometric means (95% confidence intervals) | | |
|  | 1 | 0.95 (0.91, 0.99) | 0.98 (0.96, 0.99) |
| **AST** | | | |
| N | 1,920 | 438 | 2,358 |
|  | Ratio of geometric means (95% confidence intervals) | | |
|  | 1 | 0.96 (0.94, 0.99) | 0.98 (0.97, 0.99) |
| **GGT** | | | |
| N | 1,920 | 438 | 2,358 |
|  | Ratio of geometric means (95% confidence intervals) | | |
|  | 1 | 0.95 (0.92, 0.99) | 0.97 (0.96, 0.99) |
| **Haptoglobin** | | | |
| N | 1,920 | 438 | 2,358 |
|  | Ratio of geometric means (95% confidence intervals) | | |
|  | 1 | 0.98 (0.92, 1.04) | 1.00 (0.99, 1.01) |

USS: Ultrasound scan; ALT: alanine amino transferase; AST: aspartate amino transferase; GGT: gamma- glutamyltransferase

^a^: numerator represents the number of offspring with USS fatty liver

Adjusted for offspring age at outcome assessment and gender, maternal age, parity, maternal alcohol intake, household manual social class, maternal pre-pregnancy BMI, and offspring concurrent BMI
